# Probing popular and political discourse on antimicrobial resistance in China

**DOI:** 10.1186/s41256-019-0097-z

**Published:** 2019-02-28

**Authors:** An Yi Yu, Susan Rogers Van Katwyk, Steven J. Hoffman

**Affiliations:** 1 Global Strategy Lab, Dahdaleh Institute for Global Health Research, Faculty of Health and Osgoode Hall Law School, York University, Toronto, ON Canada; 2 Faculty of Health Sciences, McMaster University, Hamilton, ON Canada; 3 School of Epidemiology and Public Health, University of Ottawa, Ottawa, ON Canada; 4 Department of Global Health & Population, Harvard T.H. Chan School of Public Health, Harvard University, Boston, MA USA

**Keywords:** Antimicrobial resistance, China, Media, Government policy

## Abstract

**Background:**

Antimicrobial resistance (AMR) is an increasing threat to global public health that is largely exacerbated by the overuse and misuse of antimicrobial medicines. As the largest antimicrobials producer and user in the world, China has a critical role to play in combatting AMR. By examining Chinese news articles and policy statements, we aim to provide an authentic understanding of public discourse in China on AMR.

**Methods:**

A search was conducted using two of the most comprehensive digital libraries for Chinese news media documents. Chinese policy documents were retrieved from official Chinese government websites. Records from June 2016 to May 2017 were included. Grounded theory was used to analyze included records, and we followed an iterative thematic synthesis process to categorize the key themes of each document.

**Results:**

Across 64 news articles, most articles delivered general knowledge about AMR and debunked AMR-related myths, explored the implications of AMR-relevant policies, and discussed the misuse of antimicrobials in the agricultural sector. All policy documents provided guidance for healthcare workers, encouraging them to better manage antimicrobial prescriptions and usage.

**Conclusions:**

While the Chinese media actively educates the public on strategies for AMR prevention, certain news articles risk misleading readers by downplaying the hazards of domestic AMR issues. Further, although several national policies are geared towards combatting AMR, the government faces difficult challenges in overcoming public misconceptions regarding antimicrobial use. Records from the regional level should also be examined to further explore China’s public discourse on AMR.

**Electronic supplementary material:**

The online version of this article (10.1186/s41256-019-0097-z) contains supplementary material, which is available to authorized users.

## Background

Antimicrobial resistance (AMR) occurs when microorganisms evolve to withstand antimicrobial medicines. This phenomenon hinders the effectiveness of antimicrobials in the prevention and treatment of a multitude of infectious diseases. Although AMR is a process that may naturally occur, the overuse and misuse of antimicrobial drugs accelerates this development [1]. AMR is now recognized to be present in every country and is said to be a serious threat to global public health [2, 3]. Given the potential threat of AMR, the World Health Organization (WHO) urges coordinated action from governments and all sectors of society to decelerate the rate of its emergence and spread [2].

Results from a survey conducted by the Chinese Academy of Sciences show that total antibiotic usage in China in 2013 was approximately 162,000 tons, which consists of 52% of usage in animals and 48% in humans, making China the largest antibiotics producer and user in the world [4]. This massive consumption of antibiotics is 150 times that of the UK in the same year [4]. Further, the consumption of antibiotics in defined daily doses per 1000 inhabitants per day (DID) was approximately six times greater than Canada, Europe, the UK and the USA [4].

The widespread use of antimicrobials in China has surely exacerbated the country’s AMR problem. Bacteria that confer resistance to colistin, a last-resort antibiotic for bacterial infections, has been identified in animals and samples from infected Chinese inpatients as of 2015 [5]. Additionally, methicillin-resistant *Staphylococcus aureus* (MRSA), which is a bacterium that may cause life-threatening infections due to its resistance, has a mean prevalence of 80.4% in Shanghai and 50.4% in China as a whole [6, 7]. These alarming figures clearly indicate that China is burdened by AMR and thus plays a crucial role in combating this global problem.

In recent years, China has joined global AMR efforts by implementing a series of national strategies to address AMR, including its National Action Plan (NAP) to Contain Antimicrobial Resistance (2016–2020) released in August 2016 [8]. The NAP has identified funding sources, a monitoring and evaluation process is in place, and the plan is currently being implemented [9]. However, the complexity of China’s health system has been a major obstacle hindering the implementation of this policy [10, 11]. The multi-layered healthcare delivery system, the heterogenous cultural and healthcare environments across Chinese jurisdictions at the provincial, city, county, town and village levels, and the profit-driven behaviour of healthcare administrators make it difficult for federal regulators that promote antimicrobial stewardship policies to be followed by all institutions [12–14]. For instance, although the state has made antibiotics a prescription-only drug since 2004, antibiotics are still commonly available in community pharmacies [10]. Partnerships between hospitals and pharmaceutical companies give healthcare providers perverse incentives to prescribe more antimicrobials, which also exacerbate the difficulty in enforcing antimicrobial stewardship [14].

Despite China’s increasing engagement with AMR, we know of no published articles that have analyzed public discourse on AMR as reflected by news media articles and policy statements in China. These original documents in Mandarin Chinese are inaccessible to most researchers who are not proficient in the language, and the lack of assessment of these records hinder many from gaining a more authentic understanding of China’s current public discourse on AMR. Combined with China’s massive domestic population and its role as a global power, there is a clear need to understand how this issue is discussed and framed in both Chinese popular discourse, as reflected by news media articles, and political discourse, as reflected in policy statements.

This study qualitatively surveys the ways in which AMR is discussed in two types of Chinese documents that are often not translated to English: news media articles and policy statements. We then discuss challenges that China faces in acting on AMR and recommend strategies for how China can strengthen its efforts against AMR.

## Methods

### Search strategy

We conducted a systematic search of news media documents in the China Core Newspapers Full-Text Database and Global Think Tanks, which are part of the China National Knowledge Infrastructure (CNKI) and Wanfang, respectively. Both the CNKI and Wanfang are digital libraries that consist of sub-databases for different types of publications, including newspapers, magazines and gazettes. For policy statements, we searched official Chinese government websites, which provide content directly from the State Council of the People’s Republic of China, the National Health Commission of the People’s Republic of China, and other relevant national-level ministries. These sources were recommended by two librarians at Nankai University in China.

All sources were searched using the following phrases in Chinese: “抗生素滥用”或者“抗菌素滥用”(“antimicrobial or antibiotic misuse”), “抗生素耐药性”或者“抗菌素耐药性”(“antimicrobial or antibiotic resistance”), and “超级细菌, 耐药细菌”(“superbugs, drug resistant bacteria”). These phrases were developed and refined by conducting pilot searches and consulting with librarians at Nankai University. We searched each phrase individually when using systems that do not offer advanced search functions, such as government websites.

### Inclusion and exclusion criteria

All records included in our analysis were published and analyzed in Mandarin Chinese. This allowed us to avoid the biases or misinterpretations that may occur in analyzing translated works. We included only records from 1 June 2016 to 31 May 2017. This one-year period was selected as it captures a period of substantial international attention on AMR and discourse on AMR. This time period follows the release of the final report by the UK Review on Antimicrobial Resistance led by Jim O’Neill [15], and captures the UN General Assembly High Level Meeting on AMR in September 2016.

Given the top-down nature of domestic policy making in China, we excluded records published by provincial- or local-level newspapers and governments and focused on national-level news articles and policies. We further excluded any news articles that reported only basic science discoveries; we did include records that discussed AMR in a societal context, such as those that emphasize the political or economic implications of AMR.

### Qualitative data analysis and synthesis

We followed a grounded theory approach to analyze included documents, which involves an inductive process of extracting and categorizing data from the records to generate new themes [16]. Prior to examining the documents, we did not form hypotheses on the type of thematic patterns that would emerge. As such, the themes that we identified are wholly based on concepts presented by the Chinese news articles and policy statements.

We conducted a three stage thematic synthesis: the first stage involved coding each document based on its key messages [17]; the second stage developed descriptive themes that connected similar articles [16]; and the third stage constructed conceptual themes that are useful for interpreting the “big picture” of the public discourse [16]. For this study, the Chinese records that matched our criteria were first read in their entirety. For each record, one researcher (AY) summarized its key points relevant to AMR in brief sentences using guidance from subheadings, topic sentences of main paragraphs, and messages that were emphasized throughout the record. These were then synthesized into fixed categories that began to reflect the main similarities across articles [see Additional file 1 for thematic synthesis details]. The categories were then explored through an iterative process and organized into conceptual themes with corresponding abbreviations.

## Results

**Fig. 1:**
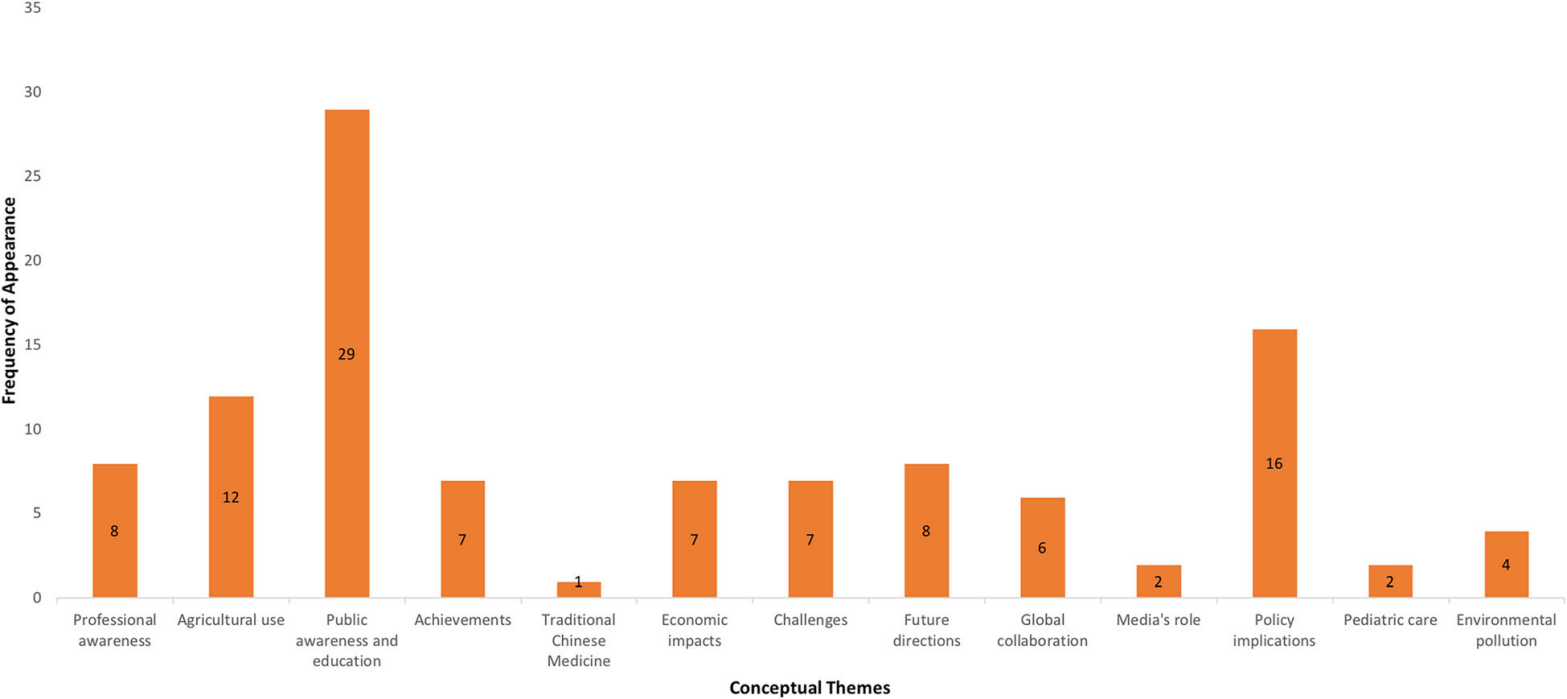
Frequency of conceptual themes in news articles

**Fig. 2:**
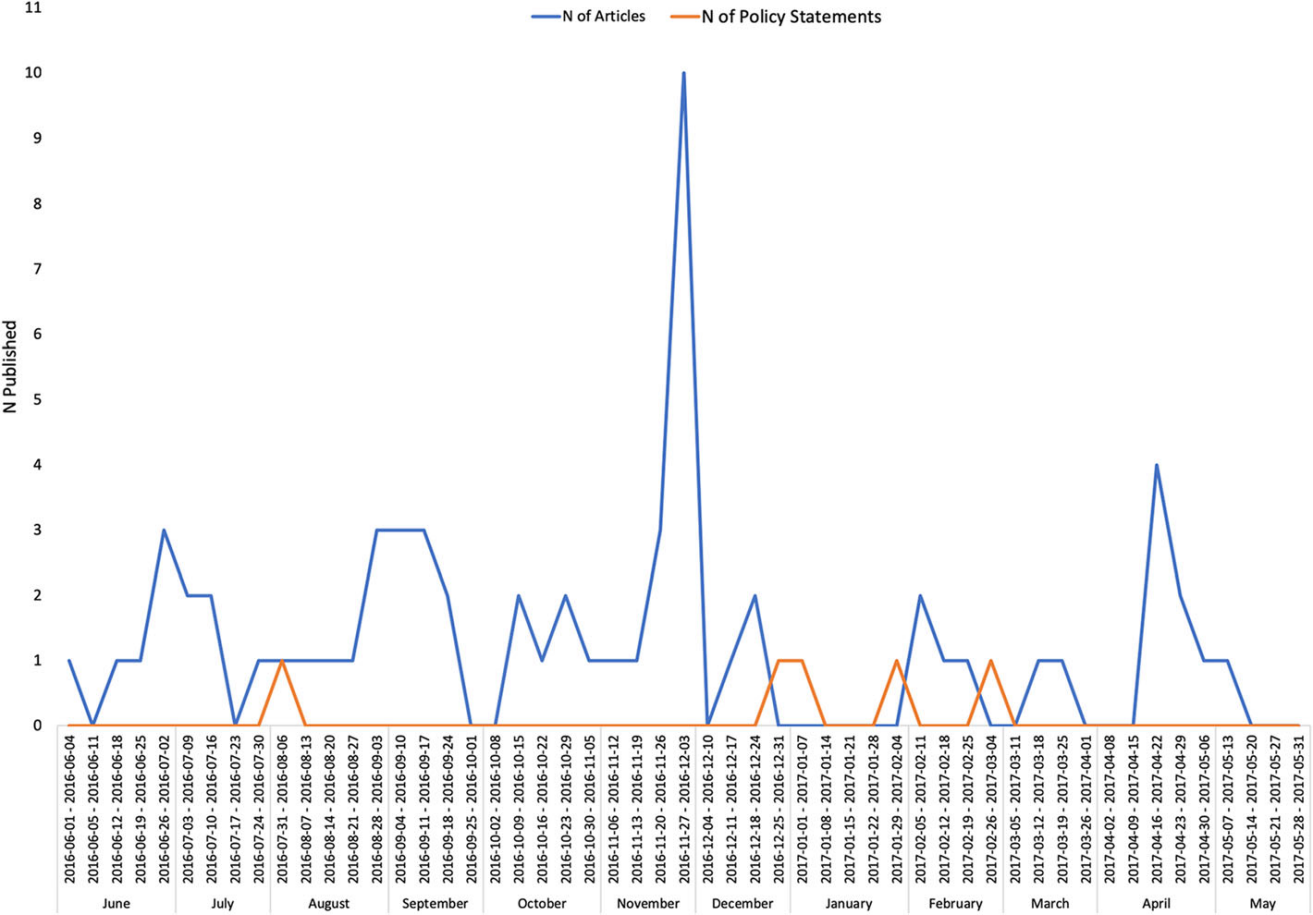
Frequency of news articles and policy statements from 1 June 2016 to 31 May 2017

We identified 114 news articles published during our study period, which was more than in the previous and following years (*n* = 100 and *n* = 61, respectively). Among the 114 news articles, 64 met our inclusion criteria and were included in our analysis, as were the five identified policy statements. Our thematic synthesis identified 14 different conceptual themes, which are described in Table 1. Figure 1 shows the frequency of these themes in news articles, with the three most prevalent themes being 1) public awareness and education, 2) policy implications, and 3) impact of agricultural use. In addition, the news media primarily addressed domestic concerns regarding AMR. Although many news articles quote recent publications from the WHO – such as the list of the world’s most dangerous superbugs [18] – only six articles were coded with the theme of global collaboration. We also examined the frequency of publications over time, which is illustrated in Fig. 2. While there was at least one news article or policy published in most weeks, the week of November 27th, 2016 was the most prolific, during which there were 10 articles published.

**Table 1:** Conceptual Themes, Ordered by Frequency of Appearance from Highest to Lowest

Conceptual Theme	Description
Public awareness and education (PAE)	Informs the public on general knowledge about AMR, how to prevent its occurrence, and debunking AMR-related myths.
Policy implications (P)	Explores implications of policies relevant to AMR. (Policies that were discussed by media articles are not limited to the policies analyzed in this study.)
Agricultural use (AG)	Emphasizes misuse of antimicrobials in an agricultural context or strategies that can address AMR in the agricultural sector.
Economic impacts (E)	Discusses any relationships between AMR, related policies, and the economy.
Challenges (C)	Discusses current challenges to addressing AMR, including those that need to be overcome to effectively implement AMR-related policies.
Professional awareness (PA)	Encourages involving healthcare providers, including physicians and administrators of healthcare institutions, to address AMR.
Future directions (FD)	Recommends future actions that the government, the public, and healthcare providers can take to address AMR.
Achievements (A)	Discusses the Chinese government’s actions or events relevant to addressing AMR in a positive light.
Environmental pollution (EP)	Frames antimicrobials as a type of environmental pollution.
Global collaboration (GC)	Focuses on China’s collaboration with the global community in addressing AMR. (Articles that only discuss recent news from the WHO without emphasizing China’s actions in the global community were excluded.)
Media’s role (M)	Emphasizes the role that media can play in addressing AMR.
Pediatric care (PED)	Focuses on how AMR impacts healthcare for children.
Traditional Chinese Medicine (TCM)	Highlights a relationship between Traditional Chinese Medicine and AMR.

### Policy statements

Similar to the news articles, most of the examined policy statements (four out of five) were aimed at China’s domestic population. The five policy documents, and eight news articles, all shared the theme of professional awareness, emphasizing that physicians and medical institutions need to better manage antimicrobial prescriptions and usage. One policy established an Expert Committee on Clinical Application and Evaluation of Antibiotic Resistance, which is tasked with providing relevant research and policy recommendations. Only the NAP – China’s most comprehensive policy targeting AMR – touches upon efforts for global collaboration and management of AMR in agriculture. The NAP also recommends promoting rational use of antimicrobials through media awareness campaigns, which closely aligns with the media’s emphasis on public awareness and education.

### News articles

The future directions presented in several news articles are also consistent with the goals and strategies stated by the NAP. The media articles provided a general introduction to AMR and the strategies recommended in the NAP to combat AMR in both healthcare and agricultural sectors, with two articles published following the release of the NAP incorporating five different AMR themes.

Many of the 29 news articles focusing on public awareness and education were aimed at counteracting the public’s fear of AMR, and quoted scientific experts to increase their credibility. However, many of these experts were unnamed and it was therefore difficult to assess the validity of their claims. Importantly, there were ten news articles that attempted to combat panic among the Chinese public that resulted when researchers found numerous antibiotic-resistant genes in Beijing’s smog [19]. Many citizens feared that these genes would directly cause severe adverse health effects in humans, but these media articles sought to assure the public that this concern is a “myth”. These articles emphasized that the bacterial genes found in the smog do not pose a major concern to public health unless they are found in viable, disease-causing bacteria, which had not been confirmed to exist in the smog by the Swedish researchers [19]. The spike in volume of media publications on AMR during the week of November 27th, 2016 can be attributed to the media’s efforts to address public concerns over the findings of the Swedish study. Several of the public awareness and education articles reassured the public of the safety of poultry consumption after wide-spread concern over antibiotics fed to chickens; these articles also touted the safety of soap that contain antimicrobials. The remaining articles with this conceptual theme focused on educating the public about basic AMR facts and how to prevent the further spread of AMR in people’s daily lives. For instance, several news articles described the global emergence of “superbugs” and urged the public to avoid overreliance on antimicrobial medicines.

We found that news articles with a policy theme discussed the challenges of implementing AMR-related policies and criticized attempts to provide a quick solution to the complex issue. One example relates to the intravenous (IV)-ban policy discussed by 11 of the 16 news articles that had a policy focus. Although it has yet to be formally established at the national level, the policy of banning all IV drug infusions at higher level outpatient clinics has been implemented in several provinces. The news articles that discussed this policy often concluded that simply banning the use of IV antibiotics is not enough to overcome AMR, as fundamental problems such as the lack of standardized physician training on antimicrobial prescribing are not being addressed. However, these articles acknowledged that stricter regulation of IV prescriptions was needed and most encouraged similar policies to be implemented across the country. China has reportedly consumed 10.4 billion IV infusion bottles in 2009, which averages to eight bottles used per citizen and is much higher than the global average [20]. More importantly, many of these infusions contain antibiotics, making them a significant contributing factor to AMR in hospitals [21]. Other policy articles touched upon the release and implementation of important policies, such as the NAP.

Twelve articles focused on the impact of agricultural use of antimicrobials on AMR and addressed concerns over antimicrobial misuse in livestock breeding. These articles also urged the government to take more specific actions to combat AMR in the agricultural sector. Two articles target the public’s discontent over antibiotic-fed chickens. Many Chinese citizens accused American fast-food chains of employing a double-standard; these companies have committed to using antibiotic-free chickens at their US stores but have yet to do so at their international stores, including those in China. The Chinese media articles we identified attempted to reassure the public that antibiotics were beneficial to public health when used responsibly during livestock breeding and that “antibiotic-free” merely means that they do not use antibiotics important in human medicine. These articles also shared the theme of public awareness and education. Additionally, many news articles claimed that the severity of antimicrobial overuse in agriculture was comparable to that in clinical settings, but only described two specific actions that the government had taken in this sector: the establishment of a new working standard to detect antibiotic levels in animal manure, and a national research project on livestock pathogen resistance and testing.

## Discussion

Our results indicate that Chinese news media articles generally aligned their focus with that of government policies, suggesting that the risks of AMR are portrayed in a way that the national government approves. Given tight media controls, we found evidence that the Chinese government likely uses its country’s news media to help manage public fears associated with health threats like AMR. In some cases, the news articles analyzed in this study appear to have employed strategies such as distorting or omitting scientific data, which may hinder Chinese citizens to readily access information that can help them confront daily issues or make informed decisions, such as whether to eat antibiotic-fed livestock. Nevertheless, Chinese news media also provides useful advice regarding AMR prevention that citizens should implement in their daily lives, such as avoiding inappropriate reliance on antimicrobials for prevention and treatment of infections when not clinically indicated. AMR prevention methods suggested by these news articles match recommendations by the WHO and reiterate the global severity of AMR [22]. In addition, we found that the news media emphasizes challenges in regulating antibiotics through the IV-ban policy. By interviewing frontline healthcare providers, the news media delivers an alternative perspective on policy implementation and allows readers to better understand the practical implications of different government policies.

### News media as an agent for managing public fear

The Chinese news media appears to be frequently used as a tool for managing public fear in the face of health threats. The Chinese media accurately depicts the consequences of AMR when it is portrayed as a global problem, but takes on a different tone when discussing contentious, internal AMR issues in China. This is unsurprising given strict media controls in China, with Freedom House ranking China last out of 65 countries for press freedom in 2015–2016 [23]. Specifically, the Chinese government uses monitoring systems and firewalls to remove articles or block websites that conflict with its political and economic agendas [23]. Of the 30 news agencies that published articles included in our analysis, only three were not run by the state or directly affiliated with a federal ministry. Thus, from a purely public health perspective, where science aligns with the government’s position, the government’s control over national media outlets represents a unique opportunity to effectively and cheaply convey helpful public health messages; but where science conflicts with the government’s position, media controls in China may pose a challenge to informing the public of health threats. Nevertheless, the news media articles analyzed in this study covered a range of issues and openly delved into the challenges that China must tackle to implement its policies.

An interesting example is the Chinese news media’s treatment of the smog myth, or the public’s fear that antibiotic-resistant genes in Beijing’s smog will cause significant harm in humans. Several international publications, including *The New York Times*, have criticized Chinese news articles that debunk the smog myth for downplaying the risks of antibiotic-resistant genes, or suggested that health advice provided by state news outlets was merely used to “assuage the worries of locals” [24–26]. *The New York Times* quotes skeptical internet users who urged the Chinese government to focus on developing strategies to combat AMR rather than reassuring the public. Internationally, there are concerns about the validity of claims made in Chinese news media articles. For instance, Joakim Larsson and his team of researchers sampled various environments worldwide for antibiotic resistance genes, and found that these genes were present in air samples from a Beijing smog event [27]. Chinese articles falsely claim that Larsson concluded that these genes are “nothing to worry about”. Upon investigation, Larsson in fact claims that the polluted city air appears to be “a more important means of transmission [of resistance] than previously thought” [27]. Nonetheless, the results from the Swedish study do not indicate whether the sampled bacteria were viable in the air and thus cannot be used to draw any definitive conclusions on the immediate health risks posed by antibiotic-resistant genes, which only foster diseases if found in live, pathogenic bacteria [19, 27]. This uncertainty is an argument repeatedly used by the ten Chinese articles addressing this smog myth to manage public fears. However, there is research suggesting that live bacteria likely exist in the air; those carrying antibiotic-resistant genes could therefore become a significant health hazard [28].

Another example of the Chinese media’s attempt to manage public fear is its response to the public’s disapproval of antibiotic-fed chickens, which are being used in Chinese restaurants. These same restaurant chains have eschewed antibiotic-fed chicken in US restaurants. Chinese news media articles may be accused of ignoring the complexity of agricultural AMR, as they emphasize that preventative antimicrobials, which can promote animal growth, are in fact beneficial to maintaining the animals’ health when used in moderation. This issue has been controversial globally, with some researchers, and members of the agricultural industry, arguing that agriculture does not significantly contribute to the development of AMR in humans [29]. Meanwhile, others assert that AMR in animals eventually spread to humans through the food chain [30]. Although Chinese news articles mainly intended to reassure that poultry in China is not unsafe compared to poultry in the US, they did mention that the amounts of preventative antimicrobials used during livestock breeding should be better regulated [see rows 3 and 53 in Additional file 1].

### Challenges in regulating IV antibiotics

The news media’s treatment of AMR was different in articles on regulating the use of IV antibiotics. Here, we found that by interviewing frontline healthcare providers, the news media delivered an alternative perspective on policy implementation, and emphasized the challenges in regulating antibiotics, thereby affording readers a better understanding of the practical implications of different government policies.

Hospitals in China are widely known to overuse IVs, even though IV is shown to have a greater risk and severity of error than other drug delivery methods [20, 31]. Several provinces in China have thus implemented an IV-ban policy, which prohibits the use of IVs in higher level outpatient clinics and appears as an efficient method to decelerate the development of AMR. However, several challenges must be overcome to successfully implement this policy, and these challenges are explored by the Chinese news media. First, the news articles analyzed in this study described the public’s persisting faith in the effectiveness of IV-delivered antibiotics for inappropriate uses. This is supported by results from a survey conducted by the WHO in 2015, indicating that 61% of Chinese participants incorrectly believe that colds and flu, which are caused by viruses, can be treated by antibiotics, which only affect bacteria [32]. Many physicians also believe that antibiotics can lead to faster recovery; many have financial incentives that motivate them to prescribe, including payments from pharmaceutical companies [33]. Thus, as several Chinese news articles described, patients who have been denied IVs at a provincial-level hospital may strongly pressure physicians to prescribe, or resort to visiting smaller hospitals that are not restricted by current IV-bans [see rows 56, 57, 60, 61 in Additional file 1]. The media also depicts logistical challenges, including the lack of standardized training of healthcare providers, as well as the lack of technical skills available in smaller hospitals to provide necessary IVs after patients are turned away by larger hospitals. In addition, negative economic impacts of the IV-ban policy pose a challenge to implementation. Policies restricting antimicrobial prescriptions have already taken a financial toll on the pharmaceutical industry, and may further discourage researchers from developing innovative antimicrobials by removing the financial incentive to do so.

#### Implications of this study

Faced with an enormous population and a complex healthcare system, the challenges identified above are difficult for the Chinese government to overcome. Nonetheless, the Chinese news media can continue to heighten public and professional awareness of AMR, as well as educate citizens about strategies that can prevent AMR’s spread. Current research efforts to identify evidence-based strategies for government interventions on AMR may support China’s efforts to protect public safety and effectively achieve the goals of its NAP [34]. The Chinese news media and government officials should probably avoid being overly optimistic about the domestic implications of AMR. Rather than using the news media to downplay AMR as it pertains to Chinese citizens, the government should undertake efforts to ensure that accurate and comprehensive scientific information is disseminated.

Based on the significant number of news articles examining the impacts of antimicrobial use in agriculture, the Chinese government could take more concrete steps towards managing antimicrobial usage in agriculture. Further, with its significant influence, China could assume a global leadership role in tackling this challenge – by role modeling progress domestically and supporting collective action globally.

Finally, despite the increase in discussions of AMR at the international level, we found that only four Chinese media articles and one policy document emphasized the need for global collaboration. As a global collective action problem, stemming the tide of AMR will require concerted action and coordination by all countries [35]. The sample of articles and policy documents reviewed here suggests China may need to develop new partnerships to adequately address AMR. Partnerships will allow the Chinese government to both provide and seek further support in implementing antimicrobial stewardship.

#### Strengths and weaknesses

This study has four key strengths. First, we conducted a systematic search of different databases and websites, capturing 365 days of activity at a time when there was significant focus on AMR globally. Second, we compared two distinct types of documents, which allowed us to analyze and compare both the government’s stated policy and the way AMR is portrayed in popular discourse. Third, we followed a rigorous qualitative methodology that involved three stages of iterative coding, and triangulation between two types of data–news media articles and policy statements. Fourth, and most importantly, we directly assessed records in Chinese rather than relying on translations, which allowed us to include many Chinese news articles and lesser-known policy statements that do not have translated versions available. These are valuable sources of information that provide a more comprehensive depiction of popular and political discourse on AMR in China.

There are three main limitations of this study. First, we analyzed articles for a one-year period; it is likely that public discourse has shifted over time, but we are unable to track long-term change in the current study. Second, we focused on public discourse at the national level only, excluding discourses in individual municipalities and provinces in China – some of which are more populous than other major countries. Different provinces in China may have unique challenges and perspectives regarding antimicrobial use that are not addressed in national-level news articles and policy statements. Third, while we searched two large databases of news-media articles and official Chinese government websites, we recognize that some media and policy sources may not be indexed in these locations, and that much of popular discourse in China occurs via web-media, such as WeChat, that may systematically differ from discourse in newspapers [36]; however, analysis of social media discourses was beyond the scope of this study. Despite these limitations, we believe that this study captures key themes of popular and political discourse on AMR in China during a significant year when AMR was high on the global political agenda.

## Conclusions

News media articles have played a constructive role in attempting to educate the public about AMR, although concerns have been raised about news articles that provide false reassurance of public health and safety. National policies reflect the Chinese government’s engagement with global action on AMR. However, efforts to implement these policies must overcome various challenges such as public misconceptions regarding antimicrobial use. Future initiatives could target agricultural AMR and global collaboration of stewardship.

To further explore Chinese discourse on antibiotics, changes or updates in popular and political discussions about AMR should be examined and compared to what is seen in other countries. Municipal and regional level documents should also be analyzed to account for the fact that China is a diverse country with many perspectives, challenges and opportunities for addressing AMR.

## Additional file

Additional file 1: Thematic Synthesis of News Media Articles and Policy Statements.xls. This MS-Excel spreadsheet provides the detailed qualitative analysis of each news media article and policy statement conducted in this study, as described in the Methods section. It also lists the source (database), publisher, publication date, access date, title, and URLs of all analyzed records. (XLSX 142 kb)

## Abbreviations

AMR: Antimicrobial resistance
IV: Intravenous
NAP: National Action Plan
WHO: World Health Organization
